# Correlation of ^99m^Tc-sestamibi uptake in renal masses with mitochondrial content and multi-drug resistance pump expression

**DOI:** 10.1186/s13550-017-0329-5

**Published:** 2017-10-02

**Authors:** Steven P. Rowe, Michael A. Gorin, Lilja B. Solnes, Mark W. Ball, Ajuni Choudhary, Phillip M. Pierorazio, Jonathan I. Epstein, Mehrbod S. Javadi, Mohamad E. Allaf, Alex S. Baras

**Affiliations:** 1 The Russell H. Morgan Department of Radiology and Radiological Science, Johns Hopkins University School of Medicine, 600 N. Wolfe St., Baltimore, MD 21287 USA; 2 The James Buchanan Brady Urological Institute and Department of Urology, Johns Hopkins University School of Medicine, Baltimore, MD USA; 3 Department of Pathology, Johns Hopkins University School of Medicine, Baltimore, MD USA

**Keywords:** Small renal mass, Renal cell carcinoma, Oncocytoma, SPECT/CT

## Abstract

**Background:**

^99m^Tc-sestamibi single-photon emission computed tomography/computed tomography (SPECT/CT) has recently been explored for the characterization of indeterminate renal masses. As judged by increased intra-tumoral radiotracer uptake, we have previously reported the excellent diagnostic performance characteristics of this test for identifying benign/indolent oncocytomas and hybrid oncocytic/chromophobe tumors (HOCTs). In this study, we investigated potential molecular mechanisms underlying the discriminatory ability of ^99m^Tc-sestamibi SPECT/CT for renal masses.

Fifty renal masses imaged with ^99m^Tc-sestamibi SPECT/CT prior to surgical resection were evaluated by immunohistochemistry for mitochondrial content and expression of the multi-drug resistance pump 1 (MDR1/P-gp). Immunohistochemical staining was scored semi-quantitatively, and results were compared across renal tumor histologies and correlated with ^99m^Tc-sestamibi uptake.

**Results:**

In total, 6/6 (100%) and 2/2 (100%) HOCTs demonstrated strong mitochondrial content staining combined with low MDR1 staining. Clear cell renal cell carcinoma showed an opposite pattern with the majority having low mitochondrial (14/26, 54%) and high MDR1 staining (18/26, 69%). Other tumor types were more variable in staining pattern, although the staining pattern reliably predicted ^99m^Tc-sestamibi uptake in almost all tumors except chromophobe renal cell carcinoma.

**Conclusions:**

Our findings confirm that renal tumors with high mitochondrial content and relatively low MDR pump expression activity accumulate ^99m^Tc-sestamibi and allow for the accurate diagnosis of the benign/indolent tumor class that includes oncocytomas and HOCTs. For masses in which MDR activity outweighs the presence of mitochondria, the tumors appear cold on ^99m^Tc-sestamibi SPECT/CT, allowing for high confidence in the diagnosis of renal cell carcinoma.

## Background

The incidence of small renal masses has increased steadily in recent decades [1]. Despite this trend, there has not been a corresponding decrease in the number of newly diagnosed cases of metastatic renal cell carcinoma (RCC) [1–3]. This discrepancy is at least partially a result of the over-treatment of benign and indolent tumors, with an estimated 5600 masses resected annually for the false presumption of cancer in the USA alone [4]. Contrast-enhanced computed tomography (CT) and magnetic resonance imaging (MRI) are unable to reliably differentiate aggressive RCC variants from benign and indolent tumors [5, 6], particularly when the mass is avidly enhancing, and the clinical question is whether the mass represents a clear cell RCC (ccRCC) or an oncocytoma (the most common malignant and benign renal tumors, respectively). Moreover, imaging with 2-deoxy-2-[^18^F]fluoro-D-glucose (FDG) positron emission tomography has also failed to show utility in differentiating between the various renal tumor histologies [7, 8].

**Fig. 1:**
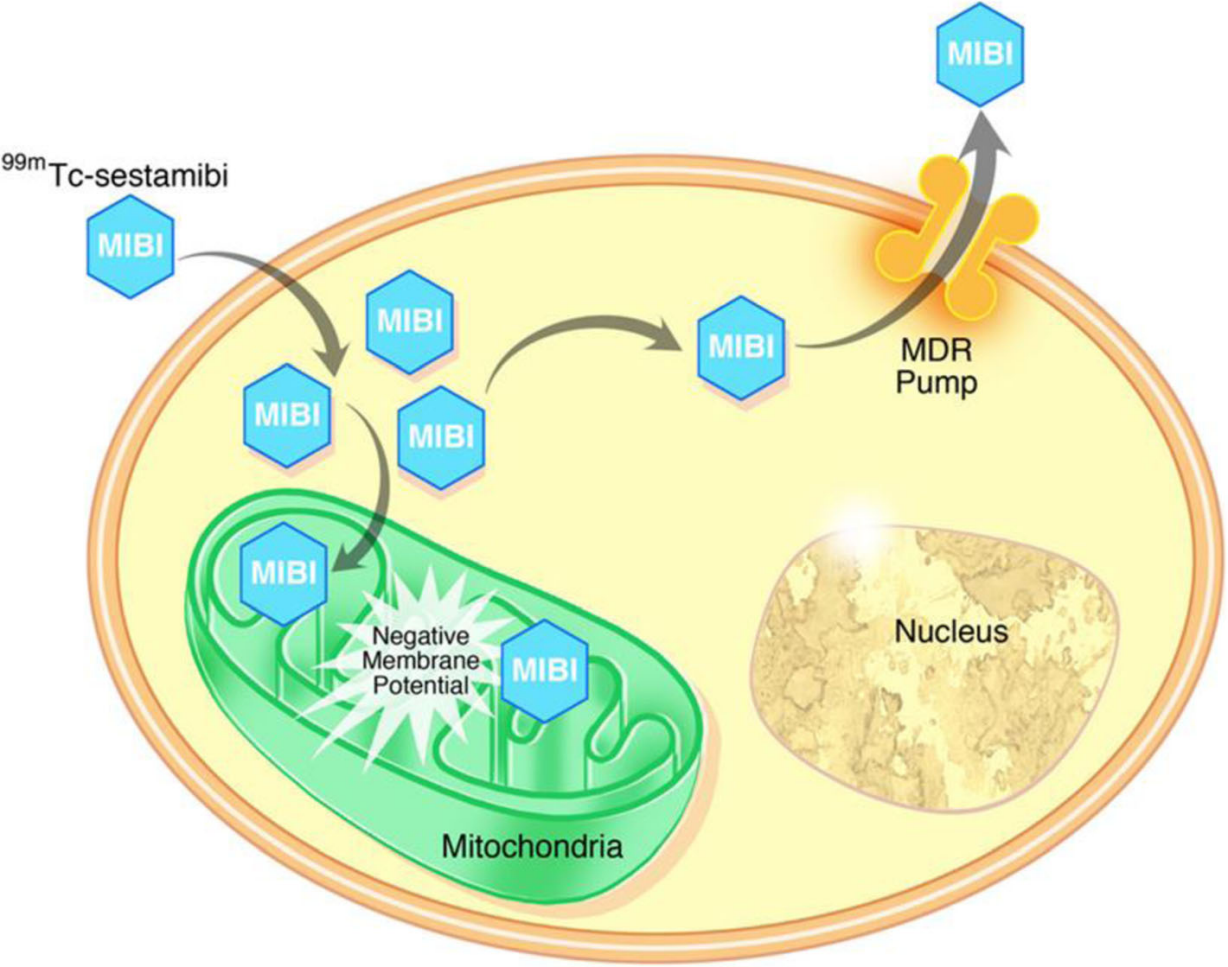
Schematic representation of the proposed cellular mechanism that explains localization of ^99m^Tc-sestamibi in renal oncocytomas and HOCTs. ^99m^Tc-sestamibi diffuses across cell membranes and is able to accumulate in cells with large numbers of functioning mitochondria on the basis of an affinity for the high negative mitochondrial membrane potential. This intracellular accumulation does not occur in cells with few or non-functional mitochondria and can be overcome by the activity of MDR pumps that actively excrete small organic molecules such as ^99m^Tc-sestamibi

Improved methods of non-invasive imaging of renal masses would allow effective risk stratification of patients presenting with these lesions [8, 9]. Our group and others have previously demonstrated the ability of ^99m^Tc-sestamibi single-photon emission computed tomography (SPECT)/CT to differentiate renal oncocytomas and hybrid oncocytic/chromophobe tumors (HOCTs) from other renal mass histologies, allowing for the non-invasive identification of these benign/indolent tumors [10–12]. The uptake of ^99m^Tc-sestamibi by oncocytomas and HOCTs is thought to derive from the high density of mitochondria in these tumors. In contrast, it is hypothesized that high expression of multi-drug resistance (MDR) pumps in tumors derived from the proximal renal tubules, such as the clear cell and papillary subtypes of RCC, overrides the presence of mitochondria and leads to relative photopenia (proposed mechanism in Fig. 1) [13, 14]. In this study, we examined the proposed mechanism of ^99m^Tc-sestamibi uptake by renal tumors through immunohistochemical analysis of mitochondrial content and expression of MDR protein 1 (MDR1/P-gp) in a series of 50 renal masses imaged with ^99m^Tc-sestamibi SPECT/CT prior to surgical resection.

## Methods

### Patients

Patients included in the present study were previously imaged as part of an institutional review board-approved prospective study of the diagnostic performance characteristics of ^99m^Tc-sestamibi SPECT/CT for imaging renal tumors. The results of the earlier study have been reported by Gorin et al. [10], and a detailed description of the patient cohort can be found in that report. In brief, 50 patients with a solid clinical T1 (≤ 7 cm) renal mass initially detected on CT or MRI were imaged with ^99m^Tc-sestamibi SPECT/CT prior to surgical resection with nephrectomy or partial nephrectomy. Surgical pathology, as determined collaboratively by two expert genitourinary pathologists (JIE & ASB), was used as the truth standard to which ^99m^Tc-sestamibi SPECT/CT was compared. The study cohort included 6 (12%) cases of oncocytoma and 2 (4%) HOCTs. The other resected renal tumor histologies include 26 (52%) ccRCCs, 8 (16%) papillary RCCs, 4 (8%) chromophobe RCCs (chRCCs), 2 (4%) clear cell papillary RCCs, 1 (2%) unclassified RCC, and 1 (2%) angiomyolipoma.

### Imaging protocol and radiographic image analysis

A detailed protocol for ^99m^Tc-sestamibi SPECT/CT imaging of renal tumors has been previously described [10]. In brief, patients were injected intravenously with approximately 925 MBq (25 mCi) of ^99m^Tc-sestamibi and were then imaged on a Symbia 16-slice SPECT/CT scanner (Seimens, Erlangen, Germany) beginning 75 min post-injection. The imaging field of view for patients with localized disease was from the lung bases through the upper pelvis. Images were iteratively reconstructed using a clinical ordered subset expectation maximization algorithm with CT attenuation correction. Acquired SPECT/CT images were exported to an XD3 workstation (Mirada, Oxford, UK) and analyzed by experienced nuclear medicine readers (LBS and MSJ) to arrive at a qualitative assessment of uptake within each tumor (i.e., “hot” or “cold”) as well as a ratio of maximum uptake in the tumor relative to the maximum uptake in the ipsilateral normal renal parenchyma.

### Tissue microarray construction and immunohistochemical analysis

Tissue microarrays (TMAs) of the 50 resected lesions were constructed utilizing 1.0-mm cores in triplicate from the same sample when possible. Antibodies for assessing mitochondrial content (Abcam; ab3298; diluted 1:750) and MDR1 expression (Abcam; ab170903; diluted 1:250) were acquired from commercial sources. A 45-min antigen retrieval (HTTR) step was performed prior to incubation with either primary antibody at room temperature for 45 min. Detection of immunolabeling was performed using anti-mouse or anti-rabbit horseradish peroxidase-conjugated secondary antibodies, and counterstaining was performed with 3,3′-diaminobenzidine. The mitochondrial stain was scored on a 4-point scale based on the percentage of cells with strong granular cytoplasmic staining: 0 (0–5%), 1+ (5–25%), 2+ (25–50%), and 3+ (50–100%). Similarly, the MDR1 stain was scored on a 4-point scale based on the percentage of cells with strong membranous staining: 0 (0–5%), 1+ (5–25%), 2+ (25–50%), and 3+ (50–100%). Both of these were scored in a blinded manner with respect to tumor type by an expert urologic pathologist (ASB). A “normalized” index of mitochondrial staining was calculated by subtracting the MDR1 staining scores from the mitochondrial staining scores (i.e., subtractive normalization).

## Results

### Immunohistochemistry

**Fig. 2:**
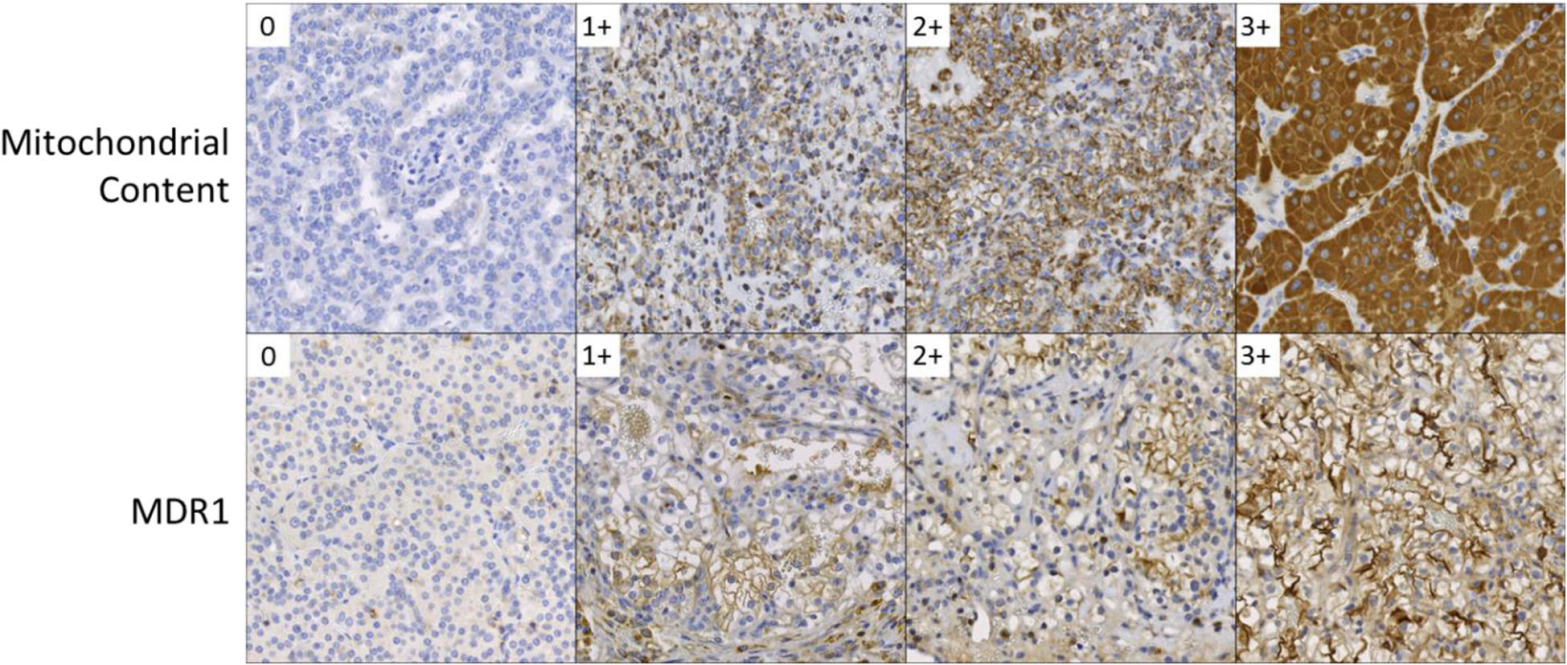
Spectrum of immunohistochemical staining for mitochondrial content and MDR1. The numbers in the white inlays within each panel indicate the IHC score of each for each representative image

Tables 1 and 2 include the semi-quantitative immunohistochemistry results categorized by tumor type for the 50 resected clinical T1 renal tumors. Representative histologic examples with differing degrees of staining are shown in Fig. 2. When examining the whole cohort, trends in the degree of immunohistochemical staining are apparent, including that all six oncocytomas (100%) and both HOCTs (100%) demonstrated 3+ staining for mitochondrial content. In contrast, 14/26 (54%) of ccRCCs had either 0 or 1+ mitochondrial staining (Table 1). The degree of mitochondrial content was more variable among the other tumor types. It is noteworthy that all of the localized cases of chRCCs had 3+ mitochondrial staining (Table 1).

**Table 1:** Semi-quantitative immunohistochemical staining for mitochondrial content stratified by tumor type

	Mitochondrial staining			
Tumor type	0	1+	2+	3+
Clear cell RCC	5 (19%)	9 (35%)	7 (27%)	5 (19%)
Papillary RCC	2 (25%)	0 (0%)	4 (50%)	2 (25%)
Clear cell papillary RCC	0 (0%)	1 (50%)	1 (50%)	0 (0%)
Chromophobe RCC	0 (0%)	0 (0%)	0 (0%)	4 (100%)
Oncocytoma	0 (0%)	0 (0%)	0 (0%)	6 (100%)
HOCT	0 (0%)	0 (0%)	0 (0%)	2 (100%)

**Table 2:** Semi-quantitative immunohistochemical staining for MDR1 stratified by tumor type

	MDR1 staining			
Tumor type	0	1+	2+	3+
Clear cell RCC	1 (4%)	7 (27%)	8 (31%)	10 (38%)
Papillary RCC	0 (0%)	0 (0%)	4 (50%)	4 (50%)
Clear cell papillary RCC	0 (0%)	1 (50%)	0 (0%)	1 (50%)
Chromophobe RCC	3 (75%)	1 (25%)	0 (0%)	0 (0%)
Oncocytoma	1 (17%)	4 (67%)	0 (0%)	1 (17%)
HOCT	1 (50%)	1 (50%)	0 (0%)	0 (0%)

Looking at MDR1 expression (Table 2), the majority (18/26, 69%) of ccRCCs had 2+ or 3+ staining. Papillary RCCs interestingly had higher levels of MDR staining than the ccRCCs with all eight cases exhibiting 2+ or 3+ staining. In contrast, five of six (83%) oncocytomas and both HOCTs demonstrated 0 or 1+ staining for MDR1. The four cases of chromophobe RCC exhibited staining behavior similar to oncocytomas and HOCTs (Table 2).

In a subsequent analysis, mitochondria staining levels were normalized by subtracting MDR1 staining scores from the observed mitochondrial staining scores. With this method of subtractive normalization, a total of 11/48 (23%) tumors had mitochondrial content staining in excess of MDR1 staining. This group included 7/8 (87.5%) oncocytomas, 2/2 (100%) HOCTs, and 4/4 (100%) chRCCs. In contrast, 37/48 (77%) tumors demonstrated levels of mitochondrial content staining that were not in excess of MDR1 staining. These 37 tumors included all clear cell, papillary, and clear cell papillary RCC in the study cohort. As demonstrated in Table 3, our method of subtractive normalization allowed for significant distinction between aggressive renal neoplasms and those with a more benign or indolent clinical behavior.

**Table 3:** Subtractive normalization of mitochondrial staining using MDR1 staining stratified by tumor histology

	Mitochondrial staining subtracted by MDR1 staining						
	− 3	− 2	− 1	0	1	2	3
Tumor type							
Clear cell RCC	0 (0%)	5 (19%)	9 (35%)	8 (31%)	4 (15%)	0 (0%)	0 (0%)
Papillary RCC	1 (13%)	1 (13%)	2 (25%)	3 (38%)	1 (13%)	0 (0%)	0 (0%)
Clear cell papillary RCC	0 (0%)	0 (0%)	1 (50%)	1 (50%)	0 (0%)	0 (0%)	0 (0%)
Chromophobe RCC	0 (0%)	0 (0%)	0 (0%)	0 (0%)	0 (0%)	1 (25%)	3 (75%)
Oncocytoma	0 (0%)	0 (0%)	0 (0%)	1 (17%)	0 (0%)	4 (67%)	1 (17%)
HOCT	0 (0%)	0 (0%)	0 (0%)	0 (0%)	0 (0%)	1 (50%)	1 (50%)

### Correlation between ^99m^Tc-sestamibi uptake and immunohistochemistry

Our previously published work on the ^99m^Tc-sestamibi uptake parameters of renal masses suggested that higher uptake ratios correlated strongly with benign and indolent histology with a ratio of 0.60 relative to background kidney allowing for accurate categorization of tumors [11]. Mitochondrial staining showed a marginal association with these ^99m^Tc-sestamibi uptake ratios (Spearman correlation of 0.24, *p* = 0.09). This association was improved by the incorporation of MDR staining via subtractive normalization (Spearman correlation increased to 0.47, *p* < 0.001). Moreover, 8/11 (72.7%) tumors that exhibited mitochondrial staining in excess of MDR1 staining also demonstrated ^99m^Tc-sestamibi uptake ratios greater than 0.6. Conversely, 36/37 (97.3%) of tumors that failed to exhibit mitochondrial staining in excess of MDR1 staining also did not demonstrate increased ^99m^Tc-sestamibi uptake ratios of greater than 0.6. Similarly, the ^99m^Tc-sestamibi uptake ratio was significantly elevated in cases in which mitochondrial staining was in excess of MDR1 staining (0.79 [95% CI 0.53–1.02] vs 0.27 [95% CI 0.23–0.32], respectively; Wilcoxon test *p* < 0.001).

**Fig. 3:**
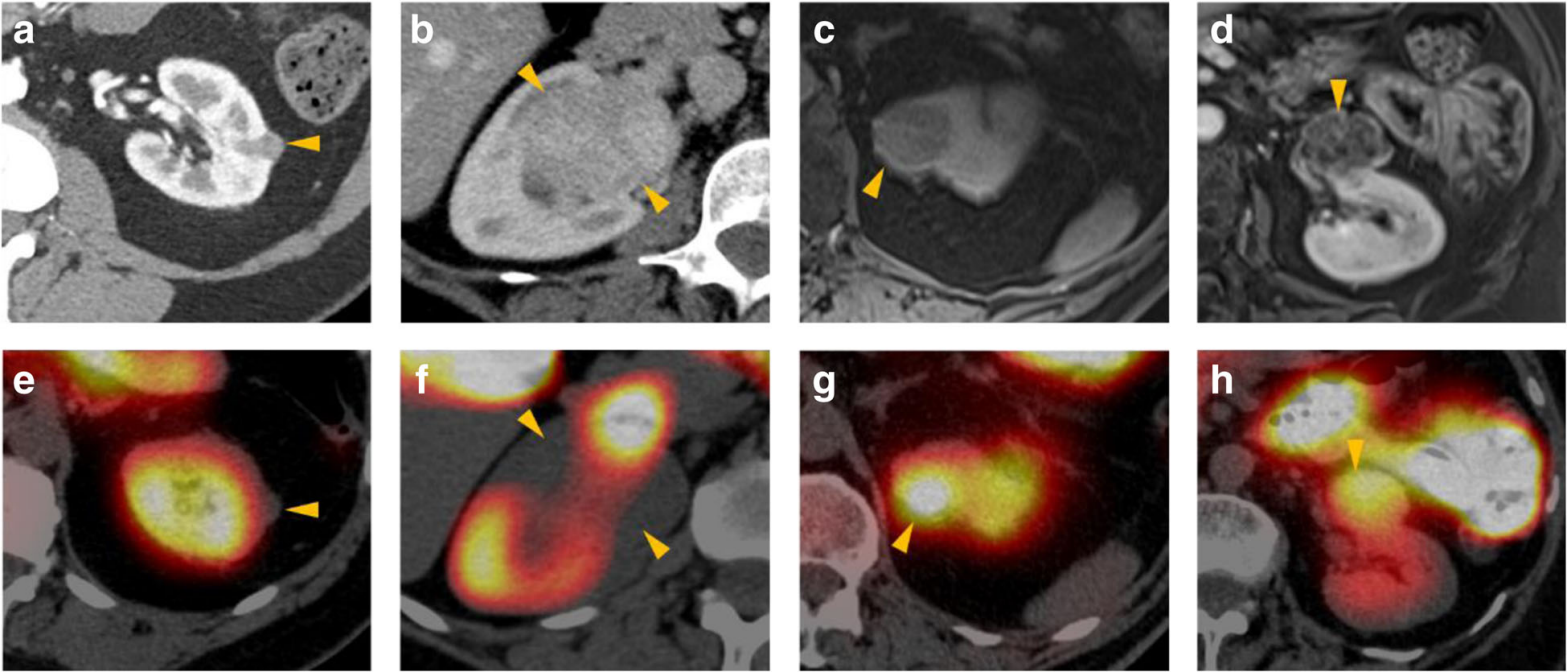
**a**–**d** Contrast-enhanced CT (**a**, **b** both axial) or post-contrast T1-weighted fat-saturated magnetic resonance imaging (MRI) (**c** coronal and **d** axial) representative images of the four chRCCs included in this study. **e**–**h** Corresponding ^99m^Tc-sestamibi SPECT/CT images of the same tumors demonstrating the highly variable degree of uptake seen in this tumor type. Tumors are denoted by yellow arrowheads. The tumors in (**a**/**e)** and (**b**/**f)** were qualitatively considered cold, whereas the tumors in (**c**/**g)** and (**d**/**h)** were qualitatively considered hot. Note the intense scatter activity from radiotracer in the common bile duct in (**f**) that must be visually excluded from the tumor to allow for accurate interpretation

The four primary chRCCs included in this series are shown side-by-side in Fig. 3, highlighting the profound visual differences in degree of radiotracer uptake in this tumor type. The chRCCs in this series demonstrated overall relatively high mitochondrial staining (Table 1) and relatively low MDR staining (Table 2), suggesting that these lesions should have high ^99m^Tc-sestamibi uptake and should visually be indistinguishable from oncocytomas and HOCTs. While this was true for 2/4 (50%) of lesions, the other two tumors were distinctly photopenic and had a visual appearance on ^99m^Tc-sestamibi SPECT/CT identical to that of ccRCC. The two hot chRCC lesions were categorized by the reviewing pathologists as the eosinophilic variant chRCC, while the cold lesions were both classical cases of chRCC.

## Discussion

^99m^Tc-sestamibi SPECT/CT is a promising new means of characterizing indeterminate renal masses that are first identified with conventional cross-sectional imaging. This immunohistochemical evaluation of 50 surgically resected tumors found that the presumptive mechanism shown in Fig. 1 accounts for the ^99m^Tc-sestamibi uptake patterns seen in the vast majority of the imaged tumors. Given the apparent utility of this imaging modality (with a published sensitivity of 87.5% and specificity of 95.2% [11]), a unified mechanism that explains the clinically observed results is reassuring that the fundamental biology of the tumors is dictating the degree of ^99m^Tc-sestamibi uptake. As such, a thorough understanding of this mechanism of radiotracer localization is desirable for a number of reasons including potential prediction of the uptake characteristics of rare subtypes of RCC that have not previously been encountered in our published cohorts (e.g., medullary and collecting duct RCC) as well as for understanding the biology of falsely positive and false negative tumors. For example, we would predict that any subtype of RCC that demonstrates high levels of MDR expression and has relatively low mitochondrial content will demonstrate photopenia when imaged with ^99m^Tc-sestamibi SPECT/CT, similar to our observations with clear cell and papillary RCC. As our knowledge in this field becomes more nuanced in regards to the patterns of uptake in different renal mass histologies, the role of other ATP-binding cassette (ABC) transporters such as MRP1 may also need to be investigated to provide a complete molecular-level understanding.

Also, this work allows insight into the irregularly false-positive nature of chRCCs in this imaging test. The similar degree of mitochondrial and MDR staining across all four cases of chRCC would have intuitively suggested that all four would have similar uptake characteristics. Indeed, all four should have been falsely positive given their relative abundances of mitochondria and low MDR expression levels. Previous work has suggested that the eosinophilic variant of chRCC has more mitochondrial genome copy numbers than normal kidney and that such tumors share a number of molecular characteristics with oncocytomas [15]. The observation that two of the imaged chRCCs were photopenic (both being classical variants) raises the possibility that more subtle ultrastructural differences underlie the variable appearance of chRCC on ^99m^Tc-sestamibi SPECT/CT imaging. Indeed, it has previously been noted in the electron microscopy literature that the mitochondria of chRCCs can have an abnormal morphology and that there may be defective mitochondrogenesis [16]. Differing concentrations of functioning versus non-functioning mitochondria in chRCC cells could explain the histopathologic and clinical findings that eosinophilic variant chRCCs demonstrate ^99m^Tc-sestamibi uptake while classical chRCCs do not.

chRCC is considered by many to be an indolent subtype of RCC with better overall and cancer-specific survival than ccRCC [17], and a recent publication on risk stratification after renal mass biopsy advocated for active surveillance of small chRCCs [18]. Rarely, however, chRCC can metastasize. To gain further insight into the application of ^99m^Tc-sestamibi imaging of this RCC subtype, we imaged two patients with metastatic chRCC (data not shown). In both patients, no ^99m^Tc-sestamibi uptake was seen within their metastatic lesions. Although it is difficult to draw conclusions from such a small patient sample size, this finding suggests that falsely positive (i.e., hot, eosinophilic variant) primary chRCCs may represent the more indolent tumor subtype and can be safely observed, whereas true positive (i.e., cold, classical variant) chRCCs may be more aggressive. Indeed, it has been reported that the eosinophilic variant of chRCC portends a better prognosis than the classical variant. [19]. If the uptake patterns we have observed in chRCCs can be born out in larger studies, this would support an even more useful role for ^99m^Tc-sestamibi SPECT/CT in the risk stratification of primary renal tumors.

## Conclusions

The degree of ^99m^Tc-sestamibi uptake in indeterminate renal masses is dictated by an interplay between the presence of mitochondria and MDR pumps. Our findings confirm that renal tumors with high mitochondrial content and relatively low MDR pump expression accumulate ^99m^Tc-sestamibi, allowing for the accurate diagnosis of benign/indolent oncocytomas and HOCTs. For most cases of RCC in which MDR activity outweighs the presence of mitochondria, lesions appear cold on ^99m^Tc-sestamibi SPECT/CT. Among the renal tumor types studied, chRCC demonstrate the most variability in ^99m^Tc-sestamibi uptake. Additional work is needed to investigate whether the uptake on^99m^Tc-sestamibi SPECT/CT corresponds to the aggressiveness of chRCC. Additional work may also be needed to examine the potential roles of other ABC transporters in better characterizing ^99m^Tc-sestamibi uptake in renal tumors.

## Abbreviations

ccRCC: Clear cell renal cell carcinoma
chRCC: Chromophobe renal cell carcinoma
CT: Computed tomography
FDG: 2-Deoxy-2-[^18^F]fluoro-D-glucose
HOCT: Hybrid oncocytic chromophobe tumor
HTTR: High temperature target retrieval
MDR: Multi-drug resistance
MDR1/P-gp: Multi-drug resistance pump 1
MRI: Magnetic resonance imaging
RCC: Renal cell carcinoma
SPECT: Single-photon emission computed tomography
TMA: Tissue microarray
